# Public sector scale–up of zinc and ORS improves coverage in selected districts in Bihar, India

**DOI:** 10.7189/jogh.05.020408

**Published:** 2015-12

**Authors:** Christa L. Fischer Walker, Sunita Taneja, Laura M. Lamberti, Robert E. Black, Sarmila Mazumder

**Affiliations:** 1 Johns Hopkins Bloomberg School of Public Health, Department of International Health, Baltimore, MD, USA; 2 Center for Health Research and Development, Society for Applied Studies, New Delhi, India

## Abstract

**Background:**

In Bihar, India, a new initiative to enhance diarrhea treatment with zinc and ORS in the public sector was rolled out in selected districts. We conducted an external evaluation to measure changes in diarrhea careseeking and treatment in intervention districts.

**Methods:**

We conducted baseline and endline household surveys among caregivers of children 2–59 months of age. We calculated summary statistics for household characteristics, knowledge, careseeking and treatments given to children with a diarrhea episode in the last 14 days and built logistic regression models to compare baseline and endline values.

**Results:**

Caregivers named a public health center as an appropriate source of care for childhood diarrhea more often at endline (71.3%) compared to baseline (38.4%) but did not report increased careseeking to public sector providers for the current diarrhea episode. In logistic regression analyses, the odds of receiving zinc, with or without oral rehydration salts (ORS), increased at endline by more than 2.7 as compared to baseline. Children who were taken to the public sector for care were more likely to receive zinc (odds ratio, OR = 3.93) and zinc in addition to ORS (OR = 6.10) compared to children who were not taken to the public sector.

**Conclusion:**

Coverage of zinc and ORS can improve with public sector programs targeted at training and increasing product availability, but demand creation may be needed to increase public sector careseeking in areas where the private sector has historically provided much of the care.

Diarrhea is a leading cause of morbidity among children under 5 years of age globally [1]. In India, nearly 150 000 children died from diarrhea in 2010 [2]. With each Indian child under 2 years of age experiencing an average of 3.1 diarrhea episodes per year [3], the need for prompt and effective treatment is great. Oral Rehydration Salts (ORS) for the prevention and treatment of dehydration due to diarrhea have been available and recommended in India since the 1980s, yet the most recent national survey reports that only 26% of children with diarrhea in the past two weeks were given ORS [4]. In India, 27% of households live below the poverty line and 69% live in rural areas, and thus successful diarrhea treatment programs will need to provide inexpensive or free treatment at the community level, either through community–based health workers or private sector doctors and retailers [4–6].

Zinc was added to the World Health Organization, UNICEF, and Indian Academy of Pediatrics’ diarrhea treatment recommendations in 2004 [7–8]. Despite national guidelines, state level adoption has been slow throughout many states in India, including Bihar. As new programs begin to incorporate zinc into routine diarrhea treatment protocols in the public and private sectors, there is an opportunity to measure program success by assessing key coverage indicators and changes in careseeking behaviors. In this paper we present the results of an external, prospective evaluation of a new diarrhea treatment initiative designed to increase the quality of care among facility and community–based public sector health workers and to improve coverage of zinc and ORS among children with diarrhea in the Indian state of Bihar.

## METHODS

### Context of evaluation

**Figure 1:**
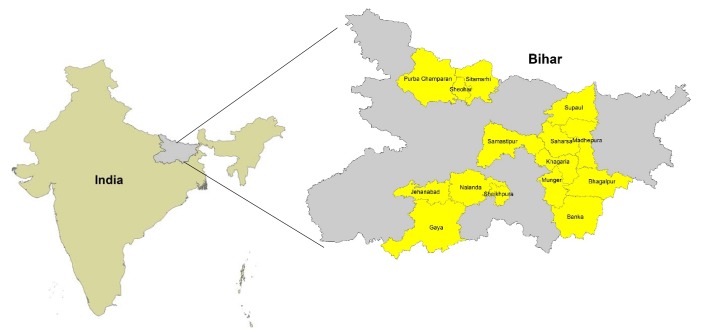
Districts included in the baseline and endline household coverage survey in Bihar.

In all15 program districts of Bihar, India (**Figure 1**), the Micronutrient Initiative (MI) led an initiative to improve diarrhea treatment quality among various cadres of public sector health care providers. MI provided training for clinic–based medical officers (MOs) and auxiliary nurse midwives (ANMs) and for community–level Accredited Social Health Activists (ASHAs) and Anganwadi Workers (AWWs). Each provider received a one–day training; the training reviewed the evidence and rationale for using zinc and ORS for diarrhea management and outlined effective strategies to counsel patients and caregivers to ensure compliance. The program also included a system of supportive supervision for the ASHAs and AWWs. To address the issue of no ORS and zinc supplies in public sector facilities, MI procured and distributed diarrhea treatment kits (DTKs), which included 14 zinc tablets and 2 ORS sachets, to public sector facilities and providers at the start of the project. The initial DTK supplies were intended to treat all cases of diarrhea among children <5 years of age for a 9–12–month period in each facility. The quantity of DTKs was estimated based on past case load. The MI kits were distributed during training and lasted until mid–2013 at which point the Bihar state government took responsibility for procuring the zinc and ORS products. The shift in product procurement and distribution was part of the original program design to ensure public sector sustainability.

The Institute for International Programs at the Johns Hopkins University Bloomberg School of Public Health and the Society for Applied Studies in New Delhi, India led the external evaluation with the objective of assessing changes in the quality and coverage of childhood diarrhea treatment in districts targeted by the public sector scale–up program (**Figure 1**). The program was focused on the public sector only, so the evaluation was heavily geared toward understanding changes in treatment behavior in that sector. We conducted two surveys among caregivers of children 2–59 months of age. A caregiver was defined as the mother or primary adult providing care for the child. We conducted the baseline survey in April – May 2011, prior to the start of training and supply distribution, to determine the pre–intervention coverage of zinc and ORS for the treatment of diarrhea among children 2–59 months of age. In order to measure changes since baseline in careseeking and treatments given to a child with diarrhea in the last 14 days, we conducted an endline survey from September – December 2013 in all intervention districts.

### Sample size calculation

We based sample size calculations for both the baseline and endline surveys on ORS coverage because zinc coverage was very low prior to the MI–led project in Bihar. To calculate the sample size required at baseline, we assumed baseline ORS coverage of 20.9% based on a previously published survey among children with diarrhea in the last 14 days [9]. In order to calculate the sample size required at endline, we assumed ORS coverage would increase from the 19.7% observed in the baseline survey to at least 28.5%. We increased the endline sample size to 750 caregivers of a child 2–59 months of age (ie, 50%) to ensure a sample big enough to demonstrate what is still an increase of public health importance. The sample size calculations for both surveys were conducted using STATA 12.0 statistical software (College Station, Texas, USA), with standard statistical assumptions (ie, two–sided test; alpha = 5%; 80% power; and non–continuity) and were increased to account for within village clustering and a 15% anticipated refusal rate [10].

### Survey procedures

All households within the selected project areas were eligible for inclusion in baseline and endline surveys. Applying seasonal two–week diarrhea prevalence to the required sample sizes, we determined that 2400 and 4995 households of children 2–59 months of age should be visited at baseline and endline, respectively. We divided the number of households equally across the 15 intervention districts because it was critical for the evaluation to have adequate representation from each of the 15 districts. For each district, we ascertained a list of villages from the 2001 census (the most recently available with village–level population data) and randomly selected villages using a population proportionate to size (PPS) sampling strategy. In each village, we limited the number of households screened for inclusion in the survey to 25 at baseline and 50 at endline. The random selection of endline villages was independent of baseline.

The survey team worked with leaders of the selected villages to divide each village into clusters of different mohallas (areas/blocks). The survey team mapped the clusters and randomly selected four from which rose to screen households for study inclusion. Starting at a central point within each cluster, the survey team moved from house to house using the right hand rule to identify caregivers of children 2–59 months of age until the total maximum sample per cluster was reached. For any house with more than one child 2–59 months, we selected the youngest child for inclusion in the survey. All caregivers were read a full consent document and provided a signature or a fingerprint (in the case of illiteracy) to indicate their willingness to participate in the survey. We interviewed caregivers and asked about household characteristics and typical careseeking practices for diarrhea. We then asked if the child had experienced diarrhea in the last 2 weeks and noted all careseeking and treatments for that diarrhea episode. We asked open ended questions and used pictures of zinc and ORS to aid in caregiver recall of treatments given.

All data collectors were from Bihar to ensure each could communicate with rural caregivers. Interviewer training, including classroom and field practice, was conducted in Bihar according to standard operating procedures. After each day of fieldwork, the survey forms were double checked by the supervisor and incomplete entries or logical errors were corrected by contacting the household immediately in person and by phone. This process ensured that all final forms were complete and free of logical errors prior to photocopying and data entry. Double data entry was completed by a trained data entry team in New Delhi.

We received ethical approval from the Johns Hopkins University Bloomberg School of Public Health Institutional Review Board and the Society for Applied Studies Ethics Review Committee in New Delhi, India.

### Statistical analysis

We calculated means, medians and proportions for household characteristics and conducted t–tests and χ^2^ tests to determine the statistical significance of differences between the baseline and endline survey populations. To compare diarrhea episode characteristics, knowledge, careseeking and treatments between baseline and endline, we built logistic regression models using generalized estimating equations (GEE) to control for within–cluster correlation (ie, village level).

We built three logistic regression models to assess the main evaluation outcome variables of zinc and ORS among children with diarrhea episode in the last 14 days. Receipt of ORS, receipt of zinc and receipt of both ORS and zinc were the dependent variables in each of the respective three models. In all models, we included round of survey (ie, baseline or endline) as the main predictor and the following variables: maternal education, sex of child age of child and careseeking. In the models of any ORS and zinc, we also included indicators of other treatment with zinc and ORS (ie, receipt of zinc was included in the regression with ORS as the dependent variable vice versa).

## RESULTS

**Figure 2:**
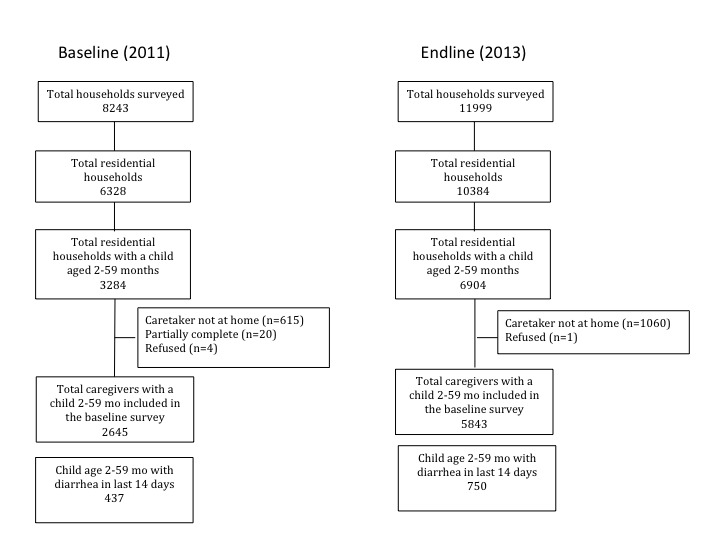
Survey profiles for both baseline and endline household surveys. mo – months.

We screened 2645 and 5843 caregivers with a child 2–59 months of age in the baseline and endline, respectively (**Figure 2**). We asked the caregiver questions about the youngest child in this age group and found the mean age of the child to be similar in the baseline (24.5 months) and endline (24.6 months) surveys. Fifty–four percent were boys in both surveys. Two–week prevalence of diarrhea was lower at the endline survey than at baseline (16.5% at baseline vs 12.8% at endline, *P* < 0.05); it should be noted that the surveys were conducted at different times of year and thus change cannot be attributed to the program. The mean age of the caregiver was 26.8 (standard deviation, SD: 5.1) years at baseline and 27.3 (SD: 5.2) years at endline, and more than 60% of mothers in both surveys had never attended school. More than half of the households included in the survey possessed a below poverty line (BPL) card and more than 80% of households had no access to an improved sanitation facility (ie, toilet or latrine). Additional household characteristics are presented in **Table 1**.

**Table 1:** Caregiver and household characteristics for children 2–59 months of age with diarrhea in the last 14 days

	Baseline n = 437 (%)	Endline n = 750 (%)	*P* value*
Mean age of child (in months)	24.5 (15.9)	24.6 (15.6)	0.916†
***Caregiver characteristics***			
Median years of father’s schooling (range)	5 (0 to 16)	5 (0 to 15)	–
Median years of mother’s schooling (range)	0 (0–15)	0 (0–17)	–
Mothers who had never been to school	292 (66.8)	477 (63.6)	0.266
Mean age of mother in years (SD)	26.8 (5.1)	27.3 (5.2)	0.940
***Household characteristics***			
Proportion of caregivers who purified drinking water	22 (5.0)	24 (3.2)	0.121
Proportion of households with water on premises or <30 min to source	436 (99.8)	740 (98.7)	0.885
Household toilet facilities:			
– Flush/pour flush to piped sewer system, septic tank or pit latrine with slab	76 (17.4)	100 (13.3)	0.055
– Pit latrine without slab/open pit	5 (1.1)	11 (1.5)	0.565
– No facility/open space/field	356 (81.5)	639 (85.2)	0.095
Proportion of households with BPL card	232 (53.1)	408 (54.4)	0.664
Religion of father/ head of the household:			
– Hindu	385 (88.1)	669 (89.2)	0.562
– Muslim	52 (11.9)	79 (10.5)	0.458
– Other	–	2 (0.3)	–
Ethnic group:			
– Scheduled caste	115 (26.3)	192 (25.6)	0.791
– Scheduled tribe	11 (2.5)	8 (1.1)	0.065
– Other backward castes	237 (54.2)	474 (63.2)	0.002
– Other	74 (16.9)	74 (16.9)	0.001

*P values generated from χ^2^–squared analysis.

†*P* values generated from t–test analysis.

**Figure 3:**
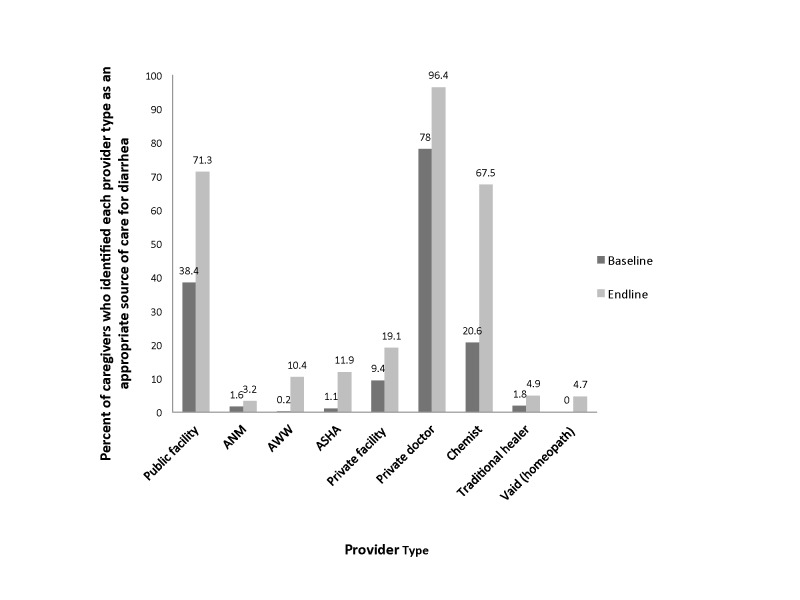
Caregiver knowledge of appropriate sources of care for diarrhea treatment at baseline and endline.

We found that caregivers were overall better able to identify a variety of providers as sources of care for a child with diarrhea at endline (**Figure 3**). However, the largest increase in awareness was found for public sector sources. At baseline, 0.2% of caregivers named an AWW as an appropriate source of care and 1.1% named an ASHA, which to 10.4% and 11.9%, respectively in the endline survey. At endline we sought to understand more about the possible shift to public sector by asking each caregiver if she had ever sought care for diarrhea treatment from these community level workers; we found that 18.9% had sought care from an ASHA or AWW for childhood diarrhea. Primary health centers (PHCs) were mentioned as an appropriate source of care by 38.4% of caregivers at baseline and 71.3% at endline; private sector sources were the most widely identified in both surveys.

The main objective of the survey was to capture caregiver careseeking and treatment for the child’s diarrhea episode in the last 14 days. Caregivers were more likely to have heard of ORS at endline than baseline (OR = 3.07, 95% confidence interval (CI): 2.32 to 4.09) (**Table 2**). More than two–thirds of children had been taken outside the home for care for the recent diarrheal episode at the time of both surveys. The relative odds of seeking care from an ASHA or AWW were much greater at endline (OR = 6.29 and 3.45, respectively). There were no differences in the proportion of children who received any treatment (82.8% at baseline vs 84.8% at endline). The odds of receiving zinc were greater at endline (OR = 3.02, 95% CI 2.17 to 4.21) compared to baseline. ORS coverage also increased at endline but more modestly (OR = 1.33, 95% CI 0.34 to 5.18).

**Table 2:** Characteristics of current diarrhea episode, careseeking and treatment practices among caregivers of children 2–59 months of age with a diarrhea episode in last 14 days

**Characteristic**	Endline vs Baseline odds ratio	95% CI*
**Clinical signs and symptoms of the child’s recent diarrhea episode:**		
Blood in stool	0.72	0.49 to 1.06
Fever	1.20	0.93 to 1.55
Vomiting	1.01	0.79 to 1.28
hirsty	0.50	0.39 to 0.65
Lethargic or irritable	1.27	1.0 to 1.63
Sunken eyes	0.87	0.68 to 1.10
Pani ki kami (local term for dehydration)	0.87	0.68 to 1.10
Proportion of caregivers who heard/seen ORS	3.07	2.32 to 4.09
Proportion of caregivers who heard of zinc	2.14	1.58 to 2.89
**Proportion of children who sought any care outside home:**		
Primary health center (PHC) / government hospital / government dispensary	1.4	0.80 to 2.53
Auxiliary nurse midwife (ANM) / sub centre	6.29	0.81 to 48.92
Anganwadi worker (AWW) / Anganwadi centre (AWC)	3.45	1.01 to 11.80
Accredited social health activist (ASHA)	2.85	0.62 to 13.06
Private sector†	1.02	0.77 to 1.34
**Proportion of children administered any treatment:**		
Syrup, unknown	0.49	0.35 to 0.64
Tablet, unknown	0.46	0.34 to 0.62
Powder, unknown	0.03	0.01 to 0.10
Injection	0.56	0.40 to 0.78
Antibiotics	2.19	1.57 to 3.06
Antidiarrheal	3.11	2.22 to 4.35
Zinc‡	3.02	2.17 to 4.21
IV fluids	1.33	0.34 to 5.18
ORS	1.42	1.07 to 1.90

ORS – oral rehydration solution, IV – intravenous, CI – confidence interval

*Logistic regression analysis using generalized estimating equations (GEE) to control for village level clustering.

†Private sector includes private doctor, hospital, chemist, or traditional healer.

‡Includes caregivers who reported zinc and those who recognized zinc on picture charts.

We conducted logistic regression analyses to identify key factors contributing to zinc and/or ORS use (**Table 3**). The adjusted odds of receiving zinc, with or without ORS, increased in the endline survey by a factor of more than 2.7 as compared to baseline. Maternal education (at least 1 year of formal schooling) was also an important predictor for zinc and/or ORS use, with adjusted odds ranging from 1.46 for ORS use alone to 2.44 for combined zinc and ORS use. Children who were taken to the public sector for care were more likely to receive zinc (OR = 3.93), ORS (OR = 5.56) and zinc with ORS (OR = 6.10) as compared to children who were not taken to the public sector. Private sector careseeking only increased the odds of receiving ORS, not zinc.

**Table 3:** Predictors of appropriate diarrhea treatment for any zinc treatment, and ORS treatment, and zinc and ORS given together

Independent variables*	Zinc		ORS		Zinc and ORS	
**OR**	**SE**	**OR**	**SE**	**OR**	**SE**
Survey conducted at endline	2.72	0.48†	1.22	0.19	2.85	0.85†
Mother has some education	2.12	0.32†	1.46	0.22†	2.44	0.56†
Age over 1 year	1.35	0.22	0.89	0.14	1.22	0.31
Female	0.97	0.15	0.80	0.12	0.96	0.22
Public sector careseeking	3.93	0.92†	5.56	1.38†	6.10	1.73†
Private sector careseeking	1.22	0.23	4.70	1.10†	2.16	1.73†
Any zinc	–	–	1.57	0.26†	–	–
Any ORS	1.53	0.25†	–	–	–	–

OR – odds ratio, SE – standard error, ORS – oral rehydration salts

*The null values for independent variables were defined as follows: baseline survey; mother reported 0 for years of school; child <1 year of age; male; caregiver did not report any public sector careseeking; caregiver did not report any private sector careseeking; caregiver did not report zinc use in open–ended question or when probed with pictures; caregiver did not report ORS use.

†*P* < 0.05.

## DISCUSSION

We conducted an external evaluation of an enhanced diarrhea treatment program conducted in the public sector in selected districts of Bihar, India. We found that in the 18 months between baseline and endline surveys, reported use of both zinc and ORS improved. We also observed that children taken to the public sector were more likely to receive zinc and/or ORS. This is not surprising given that the initiative was focused on training and supplies in the public sector and did not include training or procurement in the private sector. Though the private sector is currently treating the majority of childhood diarrhea cases, zinc had not been formally introduced into the private sector at the time of this program and evaluation, and there were no known private sector activities to promote zinc and ORS during the time of this public sector scale–up.

To ensure all zinc products used were captured at endline, data collectors were provided with additional training to emphasize the importance of asking caregivers for the packaging of all diarrhea treatments given to the child in addition to the picture charts provided to all data collectors for both surveys. Because zinc was not widely available at baseline, it is not likely that many of the reported unknowns at baseline were zinc. The additional training successfully led to an overall decrease in unknowns, however it also may have led to apparent increases in rates of antibiotics and antidiarrheals. The reduction in reported unknowns was greater overall than the combined increase in antibiotics and antidiarrheals so it is likely that much of the apparent rise in antidiarrheals and antibiotics might be the result of better identification of treatments (ie, fewer unknowns).

The evaluation was designed as a pre–post quasi–experimental design with no comparison area. This design has several limitations. Without a control group we cannot be sure all changes observed were a direct result of this initiative. However, we are unaware of any other efforts made to improve treatment quality or access to zinc and ORS in the public sector in Bihar during the period covered by these two surveys. In addition, we were not aware of any specific efforts targeting private sector zinc supplies and/or diarrhea treatment activities but did observe an increase in the number of zinc products on the market in the time between the baseline and endline surveys. It is possible that an increase in zinc available in the private sector market may have played a role in creating awareness.

We depended on caregiver recall to assess coverage of zinc and ORS for diarrhea episodes in the last 14 days and used pictures of zinc products to help caregivers recall the treatment given. Caregivers may have forgotten what treatment was given. However, if the full course of zinc was prescribed (10–14 days depending on brand), the packaging would have been available for comparison for the majority of children.

Lastly, the surveys were conducted during different seasons, which impacts diarrhea prevalence. This also could have influenced careseeking or treatments given, yet we did not see a difference in careseeking outside the home so this bias, if any, may have been minimal. Although we did not observe a significant shift in careseeking to the public sector during the course of the program, the recognition that the public sector, especially community level health workers, could be an appropriate source of care did increase. This might be considered a first step in changing careseeking behavior in the community. The public sector program was intended to improve diarrhea treatment quality and did not include demand creation activities or community level awareness activities. Therefore, the message that community health workers were now stocked with zinc and ORS for diarrhea treatment could take time to move through a community. It is possible that with time the shift will be made from awareness to careseeking. New programs might consider funding demand creation activities targeted at increasing the rate of change in the community with the hope of achieving higher community level coverage rates by increasing public sector careseeking.

With increased public sector careseeking, a renewed effort will be needed to ensure diarrhea treatment supplies are consistently in place. This initiative facilitated the availability of supplies early in the program, yet by endline only 59% of the children who sought diarrhea treatment through a public sector provider received zinc and only 50% received ORS (30% received both zinc and ORS). Lack of supplies may not be the only reason the treatments were not provided but should be considered a potential obstacle to achieving high coverage rates. If supplies are consistently problematic in the public sector, confidence in public sector care will not improve.

## CONCLUSION

Diarrhea treatment is desperately in need of an overhaul in many low– and middle–income countries [11]. Zinc for the treatment of diarrhea was incorporated into international guidelines in 2004, yet coverage of zinc remains in the single digits in most countries and ORS rates have remained stagnant for decades [8,12]. Zinc and ORS for the treatment of diarrhea are simple and inexpensive. It cannot be assumed that high coverage rates will be achieved quickly, simply by changing a policy at the national level. In the last decade, many country–level policies have incorporated zinc, but few countries have adopted comprehensive strategies to improve diarrhea treatment [13]. Achieving high coverage does not require new technology, but it does require attention to the training needs, supply logistics, and demand creation activities in both the public and private sectors.
